# Challenges in implementing continuous support during childbirth in selected public hospitals in the North West Province of South Africa

**DOI:** 10.4102/hsag.v23i0.1068

**Published:** 2018-03-22

**Authors:** Nobelungu S. Spencer, Antoinette du Preez, Catharina S. Minnie

**Affiliations:** 1 Quality in Nursing and Midwifery (INSINQ), Faculty of Health Sciences, North-West University, South Africa

## Abstract

**Background:**

According to a Cochrane review, continuous support during childbirth increases the mother’s chances of a vaginal birth without identified adverse effects. However, this evidence-based practice is not universally implemented. The objective of the study was to identify challenges encountered in implementing continuous support during childbirth in public hospitals in the North West Province of South Africa.

**Method:**

An explorative, descriptive and contextual qualitative approach was used. The data were collected during 2013 by conducting focus group interviews with 33 registered midwives who had worked in maternity units in three selected public hospitals in the North West Province for at least two years.

**Results:**

Midwives identified challenges that negatively impacted the implementation of continuous support during childbirth at organisational and interpersonal levels. At the organisational level, challenges included human resources, policies and guidelines as well as the architectural outlay of the maternity units. The personal challenges related to communication and attitudes of nurses, patients and their families.

**Conclusions:**

Organisational and personal challenges had a negative impact on the provision of continuous care during childbirth.

## Introduction

Continuous support during childbirth enhances the chances of a normal vaginal birth and according to a Cochrane review, such support has no identified adverse effects (Bohren et al. 2017). The comfort of continuous support contributes to non-pharmacological pain relief (Simkin & Bolding 2004), as it promotes the steady release of endorphins (Bohren et al. 2017). Labour support creates a feeling of security and satisfaction, enhancing positive labour outcomes (Marshall, Raynor & Nolte 2016) with a reduced risk of medical interventions such as caesarean sections (Brainbridge 2010).

Continuous support during childbirth can be provided by health professionals such as midwives (ideally, according to Aune, Amundsen and Aas 2014) or nurses, but also by doulas or laypersons, including family members. A doula is an experienced labour companion who provides emotional, informational and physical support throughout the entire birth process to the woman and her husband or partner (Medforth et al. 2009; van der Westhuizen 2011). Doulas can be appointed by the maternity unit or, in the case of a doula in private practice, by the woman herself. The current study focused on all aspects of continuous support provided by all these stakeholders.

Although best practices for continuous support during childbirth are known, they are not generally implemented, either internationally (Bohren et al. 2017) or in South Africa (Brown et al. 2007; Maputle & Nolte 2008). According to Brown et al. (2007), the level of care in South African is not ideal because most women do not benefit from the support of a childbirth companion. A South African study reported that mothers would prefer a childbirth companion, but were not informed about this possibility (Maputle & Nolte 2008).

Research on barriers impacting the implementation of continuous support during childbirth was conducted in three Arab countries (Lebanon, Syria and Egypt) (Kabakian-Khasholian, El-Nemer & Bashour 2015). Identified barriers included the absence of policies, non-optimal infrastructure of health care facilities, limited knowledge and negative attitudes of health care providers and managers. Behruzi et al.’s (2010) study, conducted in Japan, identified four groups of factors: rules and strategies, physical structure, contingency factors and individual factors. In a study conducted in Buffalo City, South Africa, Rala (2013) reported that few women knew that they could bring a companion of their choice to support them during childbirth.

The question arose as to what midwives, working in selected public hospitals in the North West Province of South Africa, perceived to be challenges in implementing continuous support during childbirth. Knowledge about these midwives’ perceptions could contribute to the formulation of recommendations for promoting continuous support during childbirth.

## Methods

Three public hospitals (one district, one regional and one large level 2 hospital) were selected to represent the different sizes of hospitals in the North West. Annually, the district hospital conducts 2270 births, the regional hospital 3100 births and the large hospital 5427 births. The population comprised all midwives working in the maternity units in the three selected public hospitals. Knowledgeable participants were selected purposefully, as they could provide rich data. The inclusion criteria specified that participants had to be registered with the South African Nursing Council (SANC) as registered midwives and should have worked in these maternity units for at least two years.

The research question was the following:

- What challenges impact the implementation of continuous support during childbirth in the selected three public hospitals in the North West Province of South Africa?

Focus group interviews were conducted from August to October 2013. At least one focus group was conducted in each public hospital until data saturation occurred. The focus groups were recorded and transcribed. Group dynamics in the groups and non-verbal communication cues were recorded as field notes (Botma et al. 2010).

Data were analysed using thematic analysis (Vaismoradi, Turunen & Bondas 2013). Verbatim quotes were linked to support the reflected themes, categories and sub-categories. The researcher and an independent co-coder analysed the same data independently and then entered into a consensus discussions to enhance trustworthiness.

## Presentation and discussion of findings

Six focus groups interviews involving 33 registered midwives were conducted. Most of the participants (88%; *n* = 29) had more than five years’ experience, were women (97%; *n* = 32), and aged 30–55 (94%; *n* = 31). Of the participants, 40% (*n* = 13) had an additional qualification in advanced midwifery.

The study revealed that midwives perceived challenges at both organisational and personal levels. At the organisational level, the identified categories related to human resources, policies and guidelines as well as the architectural outlay of the maternity units. Two identified categories at the personal level were challenges of communication and of attitudes.

The results are discussed based on Table 1. The codes at the end of the quotations each refer to the hospitals where the focus groups interviews were conducted.

**TABLE 1:** Challenges for implementation of continuous support during childbirth.

Themes	Category	Sub-categories
Organisational level	Human resources	Challenges related to midwives
		Challenges related to doulas
	Policies and guidelines	Policy regarding companions
		Guidelines regarding antenatal education
	Architectural outlay	Space
		Privacy
Personal level	Communication	Cultural differences
		Language
	Attitudes	Midwives
		Women and their families

### Theme 1: Challenges at the organisational level

The challenges regarding implementation of continuous support during childbirth are related to human resources, policies and guidelines as well as architectural outlay.

#### Category 1: Human resources

Participants emphasised that staff shortages severely limited their ability to provide continuous support during childbirth.

The first sub-category concerns challenges related to midwives. This is evident in the below quotes from the focus groups:

‘… It is definitely staff shortage that, uhm, prevents us from giving continuous care. I mean we have got, we have a lot of patients but we don’t have enough staff, so we cannot stay with that patient the whole time progressing the patient, so I think that is definitely a challenge.’ (P1)

These views of the participants in the current study seem to be confirming findings of other authors. McLachlan et al. (2011) demonstrated that maternity services worldwide are under significant strain because of increased numbers of women giving birth and critical workforce shortages, especially of practising midwives. Blaauw and Penn-Kekana (2010:24) reported a critical shortage of human resources in the public health system of South Africa, particularly midwives, advanced midwives, doctors and obstetricians:

‘If we are admitting many patients in labour rooms, we cannot just focus on that one as the others have to be helped also.’ (K2)

The ability to provide continuous support during childbirth depends on the staff-patient ratio. The National Institute for Health and Care Excellence (NICE) guidelines on safe midwifery staffing for maternity settings in the United Kingdom (2015), stressed that safe staffing implies providing one-to-one care by a midwife for every woman in established labour. One-to-one care would imply that the midwife is able to provide continuous support as she will be able to be with the woman continuously. However, even in the United States of America (USA) and in Canada, midwives usually have to attend to more than one woman in labour simultaneously (Gilliland 2011).

The midwives in this study reported that they sometimes had to attend to four patients during the active stage of labour:

‘…the staff …… you have to see of [sic] can manage two patients at the same time, but when it comes to now you have to see three patients and you are alone on that patient, the other sister is also seeing four patient [sic] alone and then there is a foetal distress in those four patients of yours and you have to go to theatre, when you come back the patient, your patient then delivered on her own [sic].’ (K2)

In the smaller district hospital, the midwives on duty were responsible for providing care to different types of maternity patients:

‘Another point is that the nurse and patient ratio is not balancing, really we are having more patients than nurses and we end up with so many complications; eh another one … the second point is that there is a shortage of staff in our hospital, really we need more staff more midwives, really there is a shortage. On night duty we have two registered midwives who are responsible for a ward of plus or minus fifty. There are labour patients, antenatal clients, post-natal deliveries, post-caesarean section, premature unit, neonatal and high and normal deliveries.’ (T1)

The participants reported that other responsibilities, such as the burden of supervision of interns, student nurses and agency nurses, could also influence midwives’ implementation of continuous support during childbirth:

‘… Most of our doctors, they are interns, they are doing their internships. They are working also in our department and the nurses are the ones helping guiding them so it is another burden on us because all responsibility will be left on the sisters and sometimes you will find the students also you know there is not always that they will have companionship from the college side or even from university. So most of the time you know development, it is only the sisters so it is an extra responsibility also on the sisters.’ (K2)

‘… The hospital make use of moonlight staff midwives, they come in and they are still inexperienced or they don’t know the environment so the care of the patient is also still at risk and then the person who is there working with them, the experienced person had also to help this people because you be there for all the patients, you have to cover for all the patients, so at the end you work the whole night.’ (K2)

The heavy workload has consequences for the midwives as well as the quality of care they can provide to women:

‘Our workload is too much for us because there is much shortage of staff, we are ending up with burnout syndrome because of this shortage of staff, and really we really do need more staff in our hospital.’ (T1)

‘They end up having burnout syndrome because of the pressure and most of them also suffer from ill health because of overloading of the work that they performed and sometimes we end up not providing quality care, especially on records; our records really are very poor. But not poor in the sense that we don’t want to eh to provide quality care but poor because we want to try but because of not enough staff we end up having poor records that could lead us to jail.’ (T1)

Igboanugo and Martin (2011) reported based on their study conducted in the Niger delta that staff shortages could affect midwives’ workload and pose a potential threat to the safety of childbearing women. In the Malawian study of Thorsen, Tharp and Meguid (2011), it was also found that burnout among health care workers contributed to poor patient outcomes or impaired performance.

If the midwife herself or another person is unavailable to provide continuous support during childbirth, the quality of care will be negatively affected:

‘And then also the companionship is not there, the relatives they are also not there to support, because if you as a midwife you are alone and there is somebody or some relative who is supporting that woman through labour that can also help; but if there is no one, then the woman becomes anxious, she pushes prematurely and all those things.’ (P1)

The staff shortage and the heavy work load can also lead to a loss of trust of the community in the midwives and the maternity service:

‘… and then I must say this has been a continuous ongoing thing of shortage of staff because we have been working like that throughout the years and yet nothing has been done and to our people in the community also don’t understand that most of the things are happening and it is due to shortage of staff. So we can’t give proper nursing care, you know, to our patients and if they have the knowledge to know that these people are working very hard maybe they could understand. In impedes really our nursing care.’ (P1)

The second sub-category under human resources concerns challenges related to doulas.

Gilliland (2011) reported that trained doulas utilise intricate and complex emotional support skills when providing continuous intrapartum support.

One of the hospitals which participated in the current study has appointed doulas, although the midwives do not think there are enough of them and that they are not optimally utilised. The other two hospitals did not have any doulas:

‘… We have no doulas available to provide continuous support…’. (T1)

The doulas are sometimes expected to do tasks not primarily part of their scope or role. Although breastfeeding and the accreditation as a baby-friendly hospital is important, it must not be seen as the doulas’ work to assist the new mothers if they are primarily appointed as doulas to provide continuous support during childbirth.

‘We as a hospital are striving for baby-friendly accreditation; so the doulas must also go to the theatre for C-sections that they can accompany the mother and baby back from theatre after the Ceaser.’ (K1)

‘The doulas are there to accompany them to the theatre and to remain with the mother, when she reaches recovery room she helps them to breast feed.’ (P1)

Even where doulas are appointed, they do not work during the nights and over the weekends:

‘At the moment they are of big help, but they are only working during the day, that is our problem they are not really working Sundays or public holidays and also night duty because they don’t get night duty allowance, they don’t get the extra money for public holidays or for Sundays.’ (K1)

#### Category 2: Policies and guidelines

The absence, formulation or lack of implementation of various policies and guidelines present challenges for the implementation of continuous support during childbirth.

The first sub-category concerns policy regarding companions.

Policies and guidelines deal with the management of pregnant patients and also specify what type of management should be provided at each level of care (Beksinska, Kunene & Mullick 2006). It can be difficult to implement continuous support during childbirth if this practice is not specifically mentioned in policies and guidelines. According to Behruzi et al. (2010), the most important barriers identified in providing humanised birth care (including continuous support) were institutional rules and strategies that restricted the presence of birth companions.

The presence of a companion is considered to be so important in Brazil that a law, the ‘Companion’s Law’, was promulgated to oblige health services to allow the presence of a companion, chosen by the parturient woman, throughout the period of labour and birth and the immediate postpartum period (Brüggemann et al. 2014).

The midwives in this study expressed the need for formalisation of the practice:

‘.. maybe we can have some policies that are implemented in the hospital to say that certain people must be able to come with this patient or to stay with this person; maybe it will make the burden of the nurses a little bit lower you see.’ (P1)

The Guidelines for Maternity Care in South Africa (South Africa. National Department of Health 2016) specify how maternity services should be rendered. These guidelines state: ‘allow family and friends to provide companionship during labour’ (p. 41) and ‘promote companionship in labour’ (p. 42) as a strategy to promote non-pharmacological pain relief. However, no provision is made in the official maternity record documents for recording the presence of such a companion.

The national guidelines are intended to be contextualised for each facility which is supposed to develop its own protocols based on the national guidelines (South Africa, National Department of Health 2016). None of the hospitals included in the current study had specific protocols regarding the presence of birth companions.

The second sub-category under policies and guidelines concern guidelines regarding antenatal education. It would be worthwhile if women are encouraged to bring their own companions along when they are in labour. Khresheh (2010) found in her study conducted in Jordan that support by a female relative is a cost-effective and beneficial practice in low-resource countries.

The Guidelines for Maternity Care (South Africa, National Department of Health 2016) do not specifically mention that women must be informed about the advantages of having a childbirth companion and encouraged to bring a companion of their choice. Participants also recommended that family members must receive information about continuous support during childbirth:

‘…even if at the clinics – the antenatal clinic – they should give proper health education to the patient, to the person, to say if this person goes into labour you must support this person, maybe she can come along with this person…’ (P1)

‘… People must be informed that this is the expectation of the person who is coming into labour room that we are expecting somebody who is going to give labour support.’ (P1)

Beksinska et al. (2006) also recommended that health education during antenatal care should inform pregnant women that they can choose to bring a childbirth companion to the maternity unit for providing continuous support to enhance the birth experience.

#### Category 3: Architectural outlay

Well-designed hospital environments, including the size and scale of hospital buildings, the layout, lighting and landscape, could impact women’s childbirth experiences (Igboanugo & Martin 2011). Internationally, the design of birth rooms is attracting much attention (Brüggemann et al. 2014; Hammond et al. 2014; Symon et al. 2013). The nurses in the study of Brüggemann et al. (2014), which was conducted in Brazil, mentioned the inappropriate physical structure of the hospital unit as a reason why they did not allow companions in their delivery rooms.

The midwives reported that the physical layout of the maternity unit posed a challenge to provide continuous support during childbirth.

The first sub-category under architectural outlay concerns space. There needs to be sufficient space for the woman and her support provider:

‘Like we are having a three bedded room here and then sometimes it is not really nice to have one patient there with a supporter and then the other one on the other curtain with the supporter. So the structure is not conducive.’ (K1)

And:

‘we will need a space for it because at the moment our space for it is not even enough and I think in most of the government hospitals the space is not enough. We find that patients are overcrowded and I think a lot has to be done when coming [sic] to infrastructure.’ (T2)

The second sub-category concerns privacy. Women in labour (and their companions) also need privacy:

‘I would say about the structure, it would be nice if like every [sic], we would have only single rooms like for privacy for all the patients like if maybe a patient is having a supporter so that they can have their own room you know.’ (K2)

Lothian (2004) emphasised the importance of privacy during childbirth and described the companion as someone who listens, watches and quietly and patiently encourages the parturient woman, making sure that she is not disturbed and has the privacy she requires.

### Theme 2: Challenges at the personal level

Lewis (2012) reported that most midwives worked hard to establish rapport with women to ensure that their experiences of care received during the childbirth process would be positive.

#### Category 1: Communication

The midwives in this research understood the importance of communication:

‘I also think that as much as we are short staffed ne, I think we have to have this relationship with the patient, continuous communication. As much as I am alone at the back, let me let the patient know I can’t be with you all the time throughout because now I am alone there are admissions there are other things that I should take care of, so I won’t uhm I won’t be able to sit next to you for the whole delivery; if maybe by also communicating with the patients maybe they will also understand because sometimes we find ourselves things will be happening, tomorrow it will be said the patient was neglected.’ (P1)

The first sub-category under communication concerns language. Participants reported communication barriers if the support provider and the woman did not understand each other’s languages:

‘We had this specific one last week here; a Shangaan and we were not able, but so luckily we were able to get an interpreter from another ward who could come and help us. And that is why sometimes it is better to get one of the family members who can also stay behind and support the patient.’ (P1)

‘Clients come from Korea, China and Pakistan, so there is a difficulty in communication.’ (T1)

Research conducted among women originally from Africa but currently staying in Australia (Murray, Windsor & Parker 2010) reported that the women’s English skills and the health providers’ ability of using interpreters impacted the effectiveness of their communications. Taniguchi and Baruffi (2007) presented similar results in that Japanese women living in Hawaii cited the language barrier as the most difficult factor during pregnancy and childbirth. Language differences also imply cultural differences.

The second sub-category concerns cultural differences. If the person providing the support and the woman in labour (and her family) are from different cultural backgrounds, then the implementation of continuous support could be a challenging task. According to Berman et al. (2008), caregivers should be culturally sensitive, culturally appropriate and culturally competent:

‘Cultural factors also are a challenge because we experience when the white patients come they usually bring a lot of people following them, even the Indian people.’ (K1)

‘Cultural factors also are a challenge because we experience when the white patients come in they usually bring a lot of people following them, even the Indian people. They are always there, they are always on your feet … and this people, especially the whites, and the Indians they are always nagging and demanding and you know wanting answers, wanting this and wanting that to be done fast and it is only three people being allocated per shift per day, night or day, but they are always in demands.’ (K2)

Taniguchi and Baruffi (2007) and Noble et al. (2009) agreed that the childbirth experience is influenced uniquely and sensitively by each culture; hence, culturally sensitive approaches would facilitate good birth outcomes and positive childbirth experiences. Noble et al. (2009) stated that caring for labouring couples required culturally competent midwives who could recognise culture-related responses to provide appropriate care to women and their babies.

Akhavan and Edge (2012) reported on a project in Sweden where immigrant women were supported by doulas who understood their culture, customs and language. The participating women reported that, in addition to support during labour, doulas provided important information and continuity of care, which apparently increased their satisfaction with and trust in maternity health care.

#### Category 2: Attitudes

If the support providers display caring attitudes, they re-assure the woman and thereby enhance the experience of continuous support during childbirth. Disrespect and abuse in maternity care are attracting much attention: both globally (Bohren et al. 2015; Bowser & Hill 2010) and in South Africa (Jewkes & Penn-Kekana 2015; Kruger & Schoombee 2010). Bad attitudes of health care providers could be considered to be a type of disrespect and abuse.

The first sub-category addressed the attitudes of the midwives. Midwives admitted that their bad attitudes could have a negative influence on their provision of continuous support during childbirth:

‘… and this shortage results in midwives attitude [sic]. They are being overworked so they become so irritable; so you know we are tired, so tired. We are irritable with our attitude - our patients; they are not convinced with the care that we are giving.’ (T1)

‘… but I also think to link it again with short staffed, because we are overworked, we are tired and we are irritated; so the staff morale is very low, so it reflects also on the patients; because we are irritated, so we get irritated with the patients; we are tired, so we [are] not nice to the patients; because we are overworked, the morale is extremely low and we are just negative.’ (K2)

Kruger and Schoombee (2010) explained the reason for the staff’s bad attitude in that they might be disempowered within the hierarchy of the medical system and feel frustrated, disappointed, resentful and even enraged in a context where they cannot be in control and cannot provide care as they might want too.

This bad attitude can have serious consequences. In addition to negatively affecting continuous support during childbirth, Igboanugo and Martin (2011) commented that the unwelcoming attitudes of staff members toward pregnant women could create social and psychological barriers, negatively affecting these women’s willingness to use health care services. The same authors maintained that poor staff attitudes could cause women to decline orthodox care and give birth at home or without skilled attendance.

The second sub-category concerns the attitudes of the women and their families. Midwives also considered patient’s attitudes to be a challenge for providing continuous support:

‘And problematic patients or problematic families you know sometimes patients eh patients from the community they come with the what, a negative attitude you know like eh they come here thinking that they are going to be shouted at, yelled at and already when they come in here they speak to us like we are nothing.’ (K1)

‘Okay it means then that it is the problematic family which are uninformed, they do not know what is expected of them; if they are stepping into labour room, then they come being misinformed at some stage and it results in negative attitude, then it inhibits the care provided to be able to achieve maintain the care towards the client which she is supposed to be getting [sic].’ (K1)

Kruger and Schoombee (2010) concluded that the reality of shortage of staff makes it impossible for midwives to always be present. In such circumstances, it seems likely that they will have to make conscious decisions as to who will receive care. This may mean that certain kinds of patients (‘difficult’, ‘disobedient’ and ‘undeserving’ patients) will systematically not be attended to. This might be true for the ‘difficult’ families too.

## Conclusions

The study participants indicated that continuous support during childbirth would be ideal but was not implemented in their maternity units because of organisational and personal challenges. The main challenges seem to be staff shortages and bad attitudes.

A midwife (meaning ‘with woman’) is the ideal person to provide continuous support (Aune et al. 2014). However, in light of the limited number of midwives and their workloads, the midwife should at least facilitate continuous support by another suitable person. All women must be encouraged to identify a person they would like to accompany them during childbirth, and ideally these persons should be orientated. In addition, a doula can be extremely valuable in supporting the woman and conditions must be as favourable as possible to enable them to perform their function optimally.

## Recommendations

### Nursing practice

- Antenatal health care providers should inform pregnant women about the benefits of continuous support during childbirth and about their choice of preferred labour companion.
- Companions should be informed about their expected role in providing intrapartum support.
- Interested community members should be identified and receive doula in-service training to provide continuous support during childbirth.
- Family members selected as companions should receive information as to how to provide continuous support during childbirth.
- A friendly, welcoming and enabling environment in labour rooms where the patient can be surrounded by her choice of preferred companion for continuous support during childbirth should be provided.
- There must be enough posts for advanced midwives, registered midwives and doulas to provide optimal care – including continuous support during childbirth.
- Agency staff should be oriented regarding continuous support during childbirth.

### Nursing education

- The importance of continuous support during childbirth should be emphasised in both the undergraduate and postgraduate midwifery curricula.
- Clinicians should also be competent in terms of communication. This goal can be achieved with cross-cultural training or the use of interpreting services to enhance continuous support during childbirth.
- The proposed curriculum content for doulas should include:
- non-pharmacological management of pain such as massage techniques, using different positions and hydrotherapy
- physical and emotional support
- cultural sensitivity.

### Policy guidelines

- Antenatal policy and guidelines should explicitly mention health education regarding continuous support during childbirth and pregnant women should be encouraged to identify a companion of their choice.
- Each maternity unit should develop a policy about the presence of persons who can provide continuous support during childbirth.
- Culturally sensitive language policies should be available to ensure that midwives work in cross-cultural situations with backup interpreters to improve continuous support during childbirth. A list of interpreters should be available in maternity units.

### Nursing research

- The following topics could enhance continuous support during childbirth:
- Developing a model of continuous support during childbirth appropriate to specific South African contexts.
- Establishing a model for patient:midwife ratios to determine how many midwives and doulas are needed in maternity units to render continuous support during childbirth.

## Limitations of the study

The study was conducted in the public sector in the North West Province, thus limiting the findings only to these hospitals. Some midwives were working in shifts, including night duty, which meant that some could not keep their focus group interview appointments. Midwives with less than two years’ experience in labour wards were excluded from the study. Their perceptions about challenges affecting the implementation of continuous support during childbirth might have added value. Only midwives participated in the focus group interviews. Consequently, women’s experiences and perceptions about continuous support during childbirth were not addressed.
